# Integrative visual analysis of protein sequence mutations

**DOI:** 10.1186/1753-6561-8-S2-S2

**Published:** 2014-08-28

**Authors:** Nadezhda T Doncheva, Karsten Klein, John H Morris, Michael Wybrow, Francisco S Domingues, Mario Albrecht

**Affiliations:** 1 Max Planck Institute for Informatics, 66123 Saarbücken, Germany; 2 University of California, San Francisco, 94143-2240 San Francisco, USA; 3 The University of Sydney, 2006 Sydney, Australia; 4 Monash University, 3145 Melbourne, Australia; 5 EURAC research, 39100 Bolzano, Italy; 6 University Medicine Greifswald, 17475 Greifswald, Germany; 7 Graz University of Technology, 8010 Graz, Austria; 8 BioTechMed-Graz, 8010 Graz, Austria

## Abstract

**Background:**

An important aspect of studying the relationship between protein sequence, structure and function is the molecular characterization of the effect of protein mutations. To understand the functional impact of amino acid changes, the multiple biological properties of protein residues have to be considered together.

**Results:**

Here, we present a novel visual approach for analyzing residue mutations. It combines different biological visualizations and integrates them with molecular data derived from external resources. To show various aspects of the biological information on different scales, our approach includes one-dimensional sequence views, three-dimensional protein structure views and two-dimensional views of residue interaction networks as well as aggregated views. The views are linked tightly and synchronized to reduce the cognitive load of the user when switching between them. In particular, the protein mutations are mapped onto the views together with further functional and structural information. We also assess the impact of individual amino acid changes by the detailed analysis and visualization of the involved residue interactions. We demonstrate the effectiveness of our approach and the developed software on the data provided for the BioVis 2013 data contest.

**Conclusions:**

Our visual approach and software greatly facilitate the integrative and interactive analysis of protein mutations based on complementary visualizations. The different data views offered to the user are enriched with information about molecular properties of amino acid residues and further biological knowledge.

## Introduction

Understanding and predicting the effect of amino acid mutations on the structure and function of a protein is still a challenging problem despite recent advances [1,2]. In the case of multiple sequence changes, it is even more difficult to distinguish the mutations with a significant effect from the ones without. Many approaches that tackle this problem have been presented in the last couple of years as reviewed in [3-8]. Computational methods such as the well-known SIFT tool [9] use evolutionary conservation derived from a multiple sequence alignment to predict that mutations of highly conserved residues have a considerable impact on function. Other methods such as the well-established PolyPhen2 tool [10] combine sequence features with structural and physico-chemical protein properties to assess the effect of a mutation. A notable disadvantage of most tools is that that they do not provide the user with a fine-grained control over the set of features used for the prediction, and the results are often difficult to interpret. In addition, those tools cannot easily cope with the speed at which new information on sequences, structures, and functions is made publicly available.

Thus, the BioVis contest selected this area of research for the 2013 data challenge. The organizers posed the question how protein function depends on the underlying protein sequence and whether it is possible to predict the effect of sequence changes. They also encouraged the use of visualization and data integration as the key to solving the problem. In particular, given the sequence of a functionally defective triosephosphate isomerase mutant (dTIM) and its parent, the yeast triosephosphate isomerase (scTIM), the task was to identify the mutations that abolish its function.

For our entry to the BioVis 2013 data contest challenge, we focused on improving the integrative visualization of a wide variety of available information on sequences, structures and functions. Our objective was to provide the biological data for a manual visual analysis and interactive exploration by the user in an integrated fashion by making it accessible through a small number of carefully designed, linked views. In this way, the user is able to generate hypotheses based on a specific view (e.g. of the protein structure) in the context of the other linked views and the provided data. As there are many biological aspects of protein sequence mutations that might affect protein structure and function, we developed visualizations that provide different levels of detail and enriched them by mapping additional data onto the graphical representations. We aimed at a generic solution that is suitable for a wide range of proteins and will support a comprehensive analysis of the impact of mutations for a large class of sequence changes. This was accomplished by a visual analytics approach integrating several software tools into a prototypic implementation freely available at the RINalyzer webpage [11].

As detailed below, we applied our approach to the data provided for the BioVis 2013 data contest. For this proof-of-concept study, we assessed the sequence changes between scTIM and dTIM by different visualizations of the protein structure together with further functional and structural information and by an exploratory analysis based on the complementary network views for both sequences.

## Methods

### General concept and views

**Figure 1:**
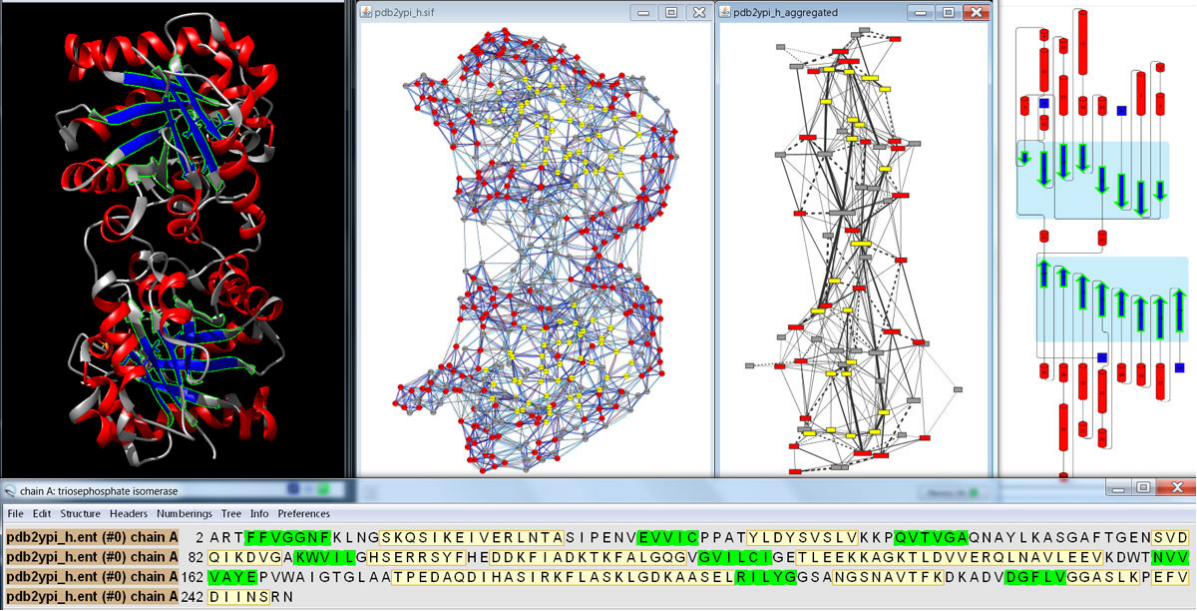
**Simultaneous visualization of biological information using different complementary views**. This overview shows the protein structure and the individual residue interactions of the yeast triosephosphate isomerase (scTIM). In particular, the three-dimensional structure and its sequence (top left and bottom, respectively) are shown with UCSF Chimera, the resulting two-dimensional view of the residue interaction network and the aggregated secondary structure network generated with RINalyzer are visualized in Cytoscape (top middle), and the cartoon image of the secondary structure elements is provided by Pro-origami (top right). Residue and network nodes are colored according to their secondary structure (strands in blue and helices in red). Strands that have been selected within UCSF Chimera are indicated by green boundary color in the structure view, by green background in the sequence view, by yellow node color in Cytoscape, and by green boundary color and blue background in Pro-origami.

To offer the available information to the user on different levels of abstraction and to support interactive synchronized exploration (Figure 1), we have carefully selected suitable visualizations as described in the following:

First, we use the standard representations of the three-dimensional (3D) structure and sequence of proteins as provided by UCSF Chimera [12,13] because sequence changes and their impact on the structure might give valuable insight. UCSF Chimera offers a variety of tools that support the interactive crosstalk between sequences and structures, affording advanced exploration of multiple sequence alignments, comparison of structures and incorporation of user-specific data. In particular, the user can study the amino acid changes between two sequences and their locations on the corresponding protein structures. It is also possible to construct a structure-based sequence alignment from the superposition of two structures. This deep integration of sequences and structures is further complemented by a multitude of molecular graphics features.

Second, we apply the RINerator tool [14] to create a two-dimensional (2D) residue interaction network (RIN) from the protein structure and visualized the RIN with the help of RINalyzer [14] within the Cytoscape platform [15]. Such a network representation is very useful to demonstrate the impact of mutations at the detailed residue interaction level by highlighting the changes of local interactions as well as long-range interaction paths, e.g. indirect interactions between residues.

Third, we offer less complex, aggregated overviews that focus on functional or structural subunits like secondary structure elements and illustrate the location and distribution of the mutations on the protein structure. In particular, we utilize the cartoon view as provided by the Pro-origami web service [16]. The main advantage of this view is that it gives a clear depiction of the chain and the secondary structure elements, while it leaves out the exact spatial location and the interrelations between those elements, which are provided by the other more detailed views. As the visual mapping from a RIN to the corresponding cartoon might be difficult for the user, a network representation that shows the RIN together with aggregated secondary structure elements can be created as an intermediate visualization.

Fourth, we extract additional structural and functional information from external databases and map these data as visual cues onto the visualizations. Functional residue annotations such as protein domain localization as well as binding and catalytic sites are important for identifying mutations that could have a direct impact on the function of the protein because they are in or near such sites. Structural properties of residues such as hydrophobicity, solvent accessible surface area, and polarity are used to characterize their potential effect on protein structure and function. Last but not least, evolutionary conservation information is crucial for distinguishing between residue changes in conserved (less tolerable of sequence changes) or variable regions.

**Figure 2:**
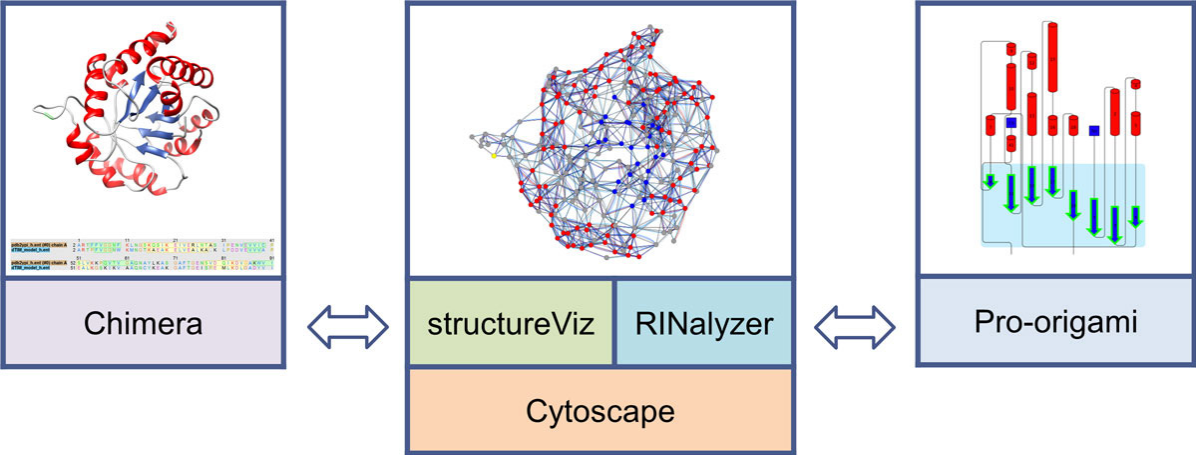
**Overview of the involved tools and the corresponding visualizations**.

Finally, the linkage between the different views is maintained by several mechanisms. Regarding the interactive exploration, we propagate the selection of elements in one view to the others. We synchronize orientation and location between RINs and structures using a special layout algorithm that we developed for this purpose. In particular, we want to ensure a consistent use of information mapping and similar cues over all views. All of the above is accomplished by adapting and extending our plugins RINalyzer [14] and structureViz [17] to integrate the freely available software tools Cytoscape, UCSF Chimera, and Pro-origami into a prototypic implementation (Figure 2). Download links and further documentation can be found at the RINalyzer webpage [11].

### RIN view and layout

**Figure 3:**
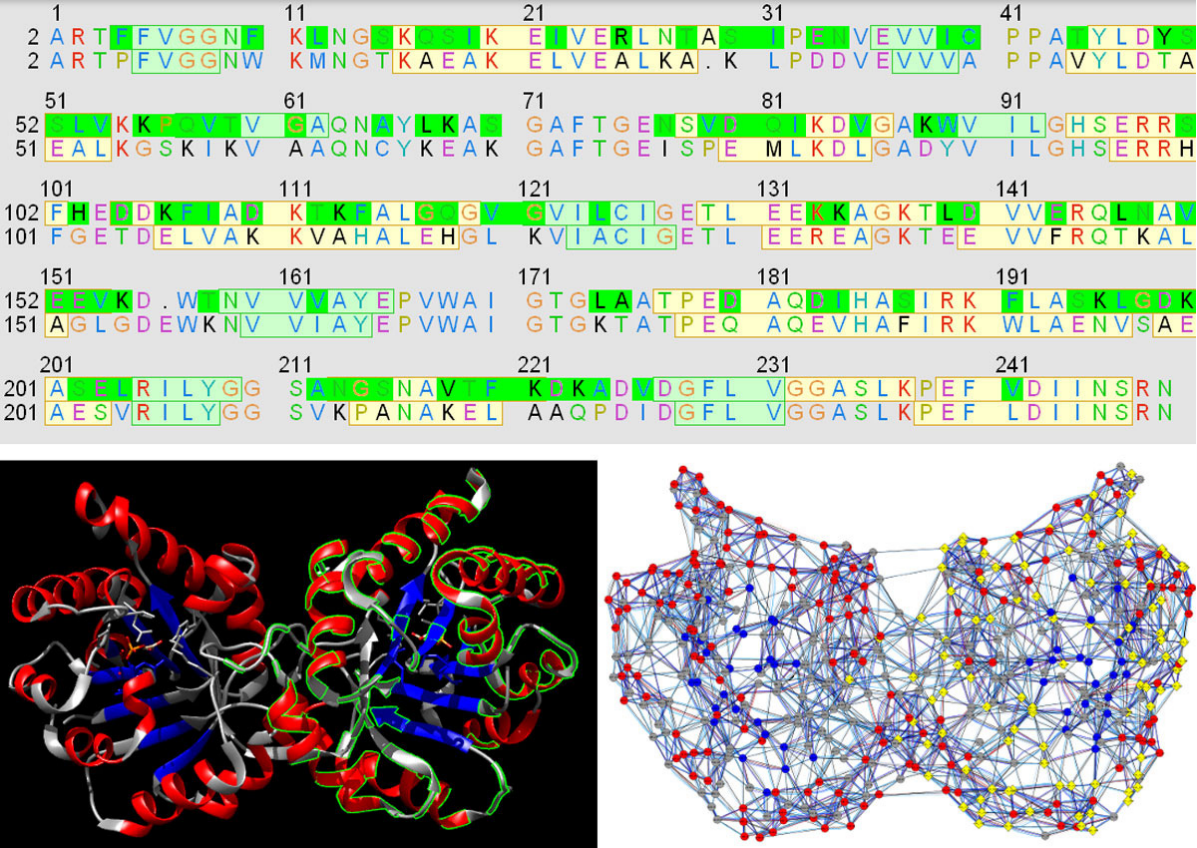
**Visualization of the sequence mutations in different views**. The alignment of the scTIM and dTIM sequences (in this order) is shown in the UCSF Chimera sequence view tool (top) and is used to identify and highlight the differences, e.g. the mutations, by green boundaries in the protein structure of scTIM (bottom left) and by yellow diamonds in the corresponding RIN view (bottom right).

The residue interaction networks (RINs) are generated by RINerator from a 3D protein structure as described previously and shown as standard network visualization within Cytoscape using RINalyzer [14,18]. In this visualization, network nodes represent amino acid residues and edges depict non-covalent residue interactions. To transfer the spatial localization information of the mutations from the structure view to the network view, we replaced the previous force-directed layout algorithm by a more appropriate stress minimization variant (Figure 1 and 3).

The new layout method is distance-based, i.e., allows specifying distances between the residues. During the layout computation, it minimizes the weighted mean square error between the given distances for pairs of residues and the geometric distance in the layout with an emphasis on local accuracy. The layout is initialized using a projection of the 3D coordinates on a 2D plane based on the UCSF Chimera view perspective. To allow for a flexible representation of the residue network and, at the same time, to preserve the user's spatial orientation using the fixed projection coordinates, we compute the stress as a balanced combination of both and increase the priority for the latter over the course of the optimization. In order to emphasize the secondary structure, the distance error weights are larger for distances between residues within the same secondary structure element. Alternatively, the layout method can prioritize certain distances based on user-defined edge weights that represent additional structural or functional information.

### Aggregated views

The aggregated views are intended to give the user a quick overview on the mutation locations with respect to specific known structural or functional regions. While it would be possible to map additional information directly onto the network representation, the RIN might become quite complex for the user. Thus, we utilize views that aggregate regions based on secondary structures, protein domain information, or functional annotations. These views serve as an intermediate visualization when switching between the 3D structure view and the 2D RIN view.

The simple cartoon view provided by the Pro-origami web service reduces the complex 3D protein structure to the essential secondary and super-secondary structure information and presents it with an easily readable layout (Figure 1). Pro-origami provides SVG images, which are enriched with further information in the form of highlighted regions of interest such as the localization of mutated residues. As Pro-origami can decompose proteins into domains, we can also obtain a combined representation of secondary structure and protein domains within the cartoon view.

### Comparison view

**Figure 4:**
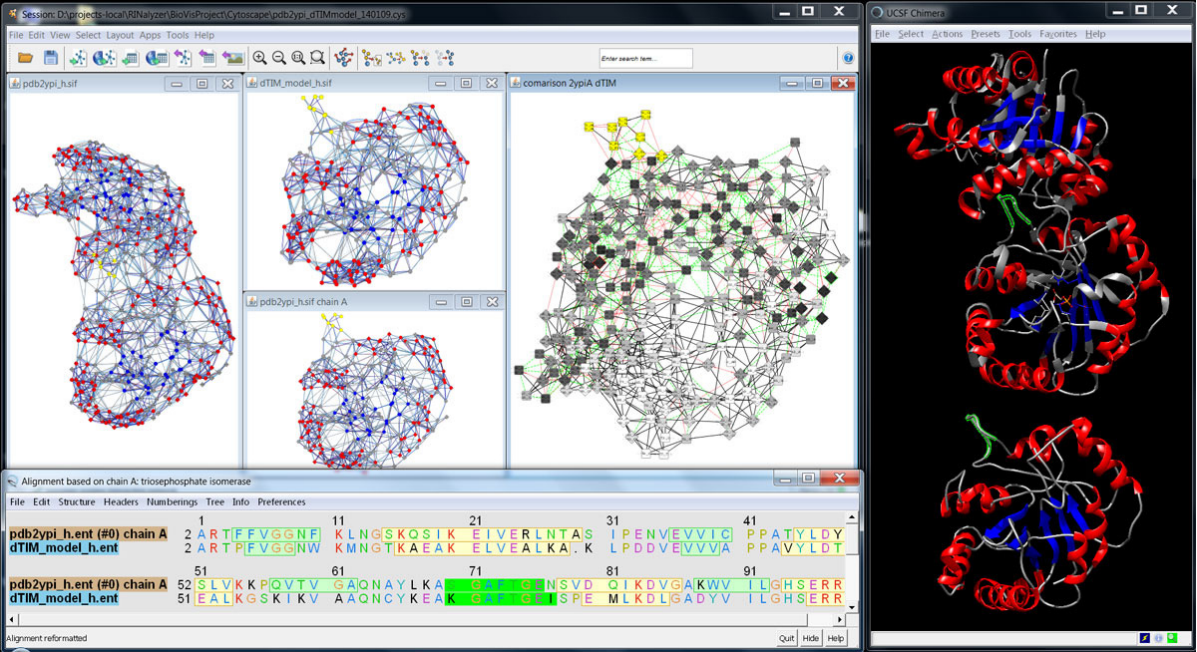
**Side-by-side views versus comparison network view**. The location of a set of residues is highlighted at the same time in all views, from left to right, the RIN of the 3D structure of scTIM, the RIN of the model of dTIM as generated by the SQWRL web server, the RIN of chain A of scTIM, the comparison RIN, the sequence alignment of the scTIM and dTIM sequences, and the corresponding 3D structures. The network nodes and residues are colored according to secondary structure (strands in blue and helices in red), except for the comparison RIN, where the nodes are colored according to the fraction of adjacent interaction edges that do not change upon mutation (from white for all to gray for none). Selected nodes are shown in yellow color in the network views and with green boundary or green background in the structure and sequence view, respectively. Such a combination of views allows the user to study the structures and networks side-by-side or all at once in the comparison network.

The representation of protein structures as RINs enables network comparison and alignment to explore the differences between parent and mutant structures further. Besides the comparison of two networks or structures side-by-side, we provide a comparison network view based on the alignment of the underlying sequences (Figure 4). In this view, each node represents a pair of aligned residues and two nodes are connected if the corresponding residues have a non-covalent interaction in either of the two compared RINs. We use visual cues to highlight interactions that were gained or lost upon amino acid change, and we score the fraction of such interactions for each residue to quantify the mutational effect on protein structure and function.

Furthermore, to distinguish more or less likely mutations, we integrated the amino acid substitution scores from the Blosum62 matrix [19] in RINalyzer and assigned a score to each mutated residue in the comparison network. Each score can be used to highlight sequence changes with a stronger impact on the protein.

### Data enrichment

**Figure 5:**
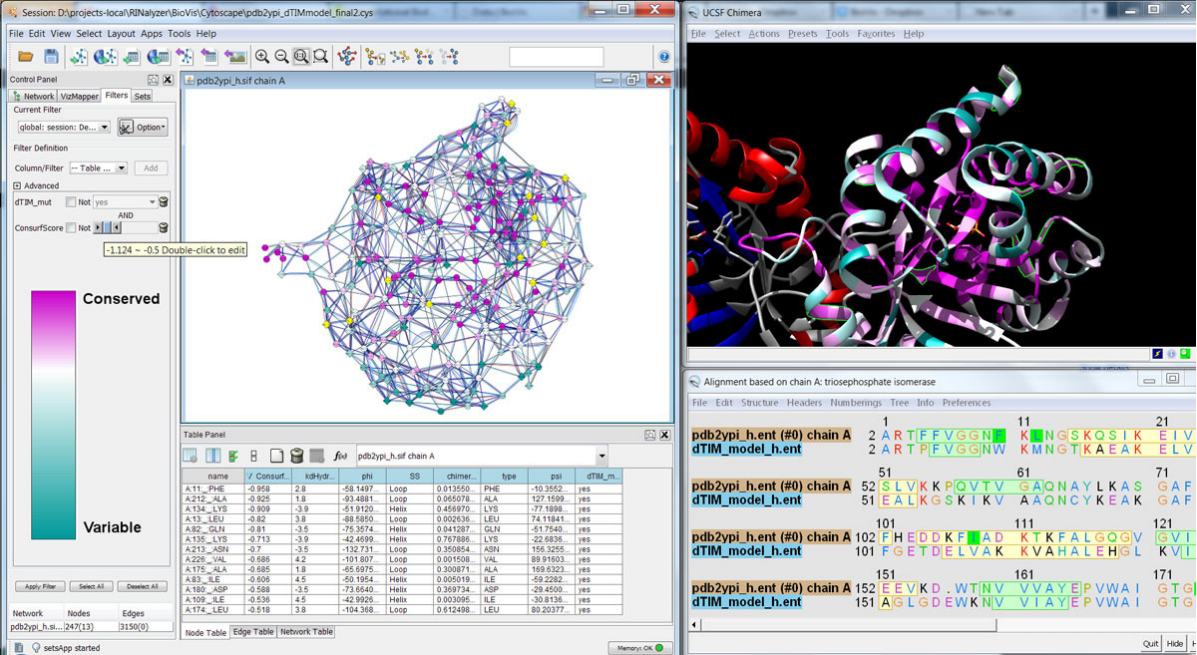
**Mapping of conservation information onto the sequence, structure, and network representations**. The nodes and residues in the RIN (top left) and chain A of scTIM (top right) are colored according to the conservation scores retrieved from Consurf-DB (turquoise-to-pink coloring indicates variable-to-conserved sites). The network nodes that represent mutated residues with a high conservation score (F11, L13, Q82, I83, I109, K134, K135, L174, A175, D180, A212, N213, V226) are selected using two filters in Cytoscape (left) and highlighted in the network view by yellow color (top left) and in the other two views by green boundary around the structure (top right) or the amino acid letter (bottom right). Nodes that correspond to mutated residues are depicted as diamonds. Additional data annotated to the residue nodes is shown in the Cytoscape attribute browser as table (bottom left).

An important component of our visual analytics approach is the mapping of available knowledge onto the visualized sequences and structures. The availability of this information in an easily accessible way while the user works with the different views should facilitate the biological knowledge discovery considerably. This is accomplished by importing the relevant data as node attributes in Cytoscape, which automatically associates them with the RIN and the protein structure. An additional benefit of this integration is that it enables the use of the built-in Cytoscape functionality to create filters based on the imported data and to highlight the residue nodes with attribute values within a given range, e.g. with high or low conservation scores (see Figure 5).

Therefore, in addition to the data given in the contest, we generated or retrieved data from multiple external sources to enrich our visualizations. The following information is regarded as potentially useful for protein analysis:

• Family conservation. ConSurf-DB [20] provides pre-computed profiles of evolutionary sequence conservation.

• Residue interactions. The RINerator package creates a network of noncovalent residue interactions such as contacts and hydrogen bonds for any 3D protein structure.

• Functional sites. Active and binding site information is retrieved manually from UniProtKB [21].

• Domain annotation. Protein domain information is obtained from the SCOP [22] online resource.

• Structural properties. Data for the solvent accessible surface area, secondary structure, hydrophobicity, and other structural properties is retrieved automatically from UCSF Chimera.

### Visual cues

The data used to enrich our visualizations is mapped as visual cues like color, shape, or line stroke in the network view and transferred to the other views where possible. Furthermore, the differences caused by the mutations can be highlighted by such cues in all visualizations.

We decided to control most visual properties via user-adjustable options with reasonable defaults. For example, different node shapes are used to distinguish the mutated residues in both the parent and the defective protein (Figure 3). Additionally, several visual styles are offered that map different functional and structural information on the views so that the user sees the distribution of corresponding values for the whole protein. Dark colors usually correspond to significant values such as strong hydrophobicity, large solvent accessible surface area or high number of changed residue interactions (Figure 4). For evolutionary conservation, the pink-to-turquoise coloring as applied by ConSurf-DB is used (Figure 5).

The visual cues are particularly useful for illustrating the changes in residue interactions due to the mutations in the comparison network view generated from the alignment of the respective sequences in UCSF Chimera. Residue interactions that are either lost or gained upon mutation are highlighted by differently colored and shaped lines (Figure 4). Residues that cannot be aligned are depicted by nodes with different node borders.

### Linkage and coordination of views

To ease the user's cognitive load when switching between different views and tools, we link them in multiple important ways. For an interactive exploration, we implemented a global selection concept, that is, the selection of elements in one view leads to the immediate selection of their corresponding representatives in all other views. Our linkage concept also ensures the consistent use of information mapping and similar cues over all views, particularly, regarding the usage of colors.

Further coordination is achieved due to the synchronized orientation and location of the graphical representations in the different views. For instance, the user can freely explore the 3D structure within the UCSF Chimera window, e.g. by rotating the protein structure. The network view can then be adjusted according to the new orientation of the rotated structure by applying the 3D-structure based RIN layout described above.

In order to implement the full linkage between Cytoscape and UCSF Chimera, we made use of their new software versions. We also ported the plugins RINalyzer and structureViz to work with Cytoscape 3, which also allowed us to link them closely. For example, while the direct communication between Cytoscape and UCSF Chimera is handled by structureViz, the structure-based layout algorithm is implemented in RINalyzer and invokes structureViz to retrieve the current spatial coordinates.

## Results and discussion

### Visual analytics approach

Our visual analytics approach assists the user's reasoning about the biological impact of mutations by interactive visualizations of sequence and structure information enriched with additional biological knowledge such as evolutionary sequence conservation and functional annotations. To show the different aspects of the data, we combine the well-known 3D structure view and the one-dimensional sequence view with the 2D RIN view. In addition, we create simplified network representations to enable the user to focus on certain biological aspects, e.g. protein domains, secondary structure elements, and functional annotations.

Besides the sequence that is given as input, a variety of information is available that can be used to interpret the functional effects of sequence changes. This includes sequence conservation, which might point to highly conserved regions responsible for some function, protein domain information, functional annotations (e.g. on molecular binding), structural properties such as hydrophobicity and solvent accessible surface area, and already known mutations and their impact. We incorporate a number of sources for such information in our approach as described above and map the data mainly as visual cues on top of the graphical representations of the protein structure and the RINs. In addition, we make use of the network representation provided by RINalyzer as well as the Cytoscape analysis capabilities to facilitate data exploration by filtering and combining the available information on individual residues.

Furthermore, to present sequence changes on the structure and residue interaction level simultaneously, we provide both a single cumulative view and two separate views of the parent and the defective mutant side-by-side. While a single view facilitates the identification of changed sites, the dual view solution allows the user to identify the structural impact of the changes, for example, lost residue interactions might alter the protein structure.

**Figure 6:**
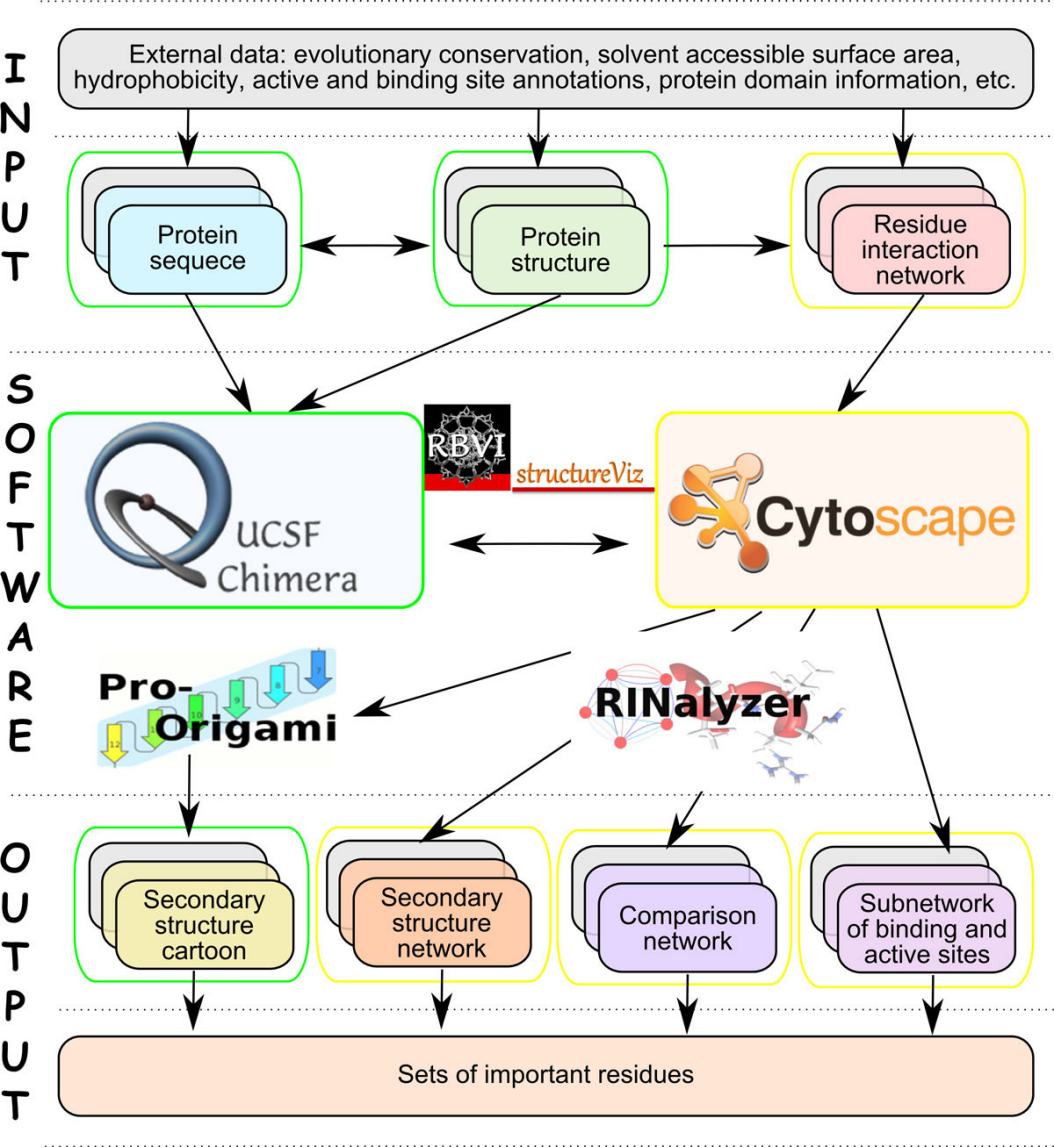
**General analysis workflow**. The workflow consists of three parts: input, software and output. The input consists of biological data, which might be protein sequences, structures, RINs as well as additional annotations and biological knowledge retrieved from external sources and databases (shown as gray background for each view). The middle part of the workflow shows the interactions between the different tools and which tool is responsible for the presentation of which data. The output consists of the different views with data mapped onto them and sets of important residues that can be identified through visual exploratory analysis of the available data. The yellow and green boundaries indicate the default selection color used by the different tools.

A general analysis workflow is presented in Figure 6. Normally, the user starts with one or more experimentally determined protein structures and retrieves or generates RINs for them. In case only sequences are available, external tools for predicting the 3D structure could be used instead. External data such as evolutionary conservation and functional annotations need to be prepared in a format compatible with Cytoscape and the RIN specifications. Then the data is loaded by the user into Cytoscape and UCSF Chimera. Further views such as the secondary structure cartoon, the aggregated secondary structure network or the comparison network can be created from within Cytoscape. The sequences of the structures can be displayed and manipulated from within UCSF Chimera. Functional annotations and evolutionary conservation have to be imported manually into Cytoscape as node attributes of the RINs, while structural properties can be retrieved automatically from the protein structures currently opened in UCSF Chimera. These data can then be applied to create the visual cues and semi-automatically propagate them to the different views. Finally, by browsing and filtering the data in Cytoscape and UCSF Chimera, the user can identify relevant amino acids, in particular, mutated residues with a potentially strong effect on the protein function. Even if the visual analysis does not immediately reveal the functional consequences of mutations, our software will provide the user at least with very useful biological indications for the molecular analysis and further experiments.

### Contest use case

In the following, the effectiveness of our integrative visual analytics approach is illustrated with the help of a typical use case based on the data provided for the BioVis 2013 data contest. For the specific case in which a functionally defective dTIM sequence is given together with its yeast scTIM parent sequence and structure, we perform a comprehensive assessment of the structural and functional impact of the sequence mutations and highlight the differences between the sequences in complementary views.

For scTIM, we retrieved the 3D structure from the RCSB Protein Data Bank [23] [PDB:2YPI] and downloaded the precomputed RIN from the RINdata web service [14]. Since there is no experimentally resolved protein structure of dTIM, we used the SCWRL Server [24] at BIC-JCSG with default settings and the parent structure as template to generate a three-dimensional model. A RIN for the defective mutant was created from the modeled structure by our RINerator package.

External data such as functional annotations, conservation information and structural properties was parsed and imported as attributes in Cytoscape to allow for mapping the data as visual cues on the network and structure views. The UCSF Chimera sequence tool was used to view, align and explore the parent and defective TIM sequences. Based on the sequence alignment, the nodes representing mutated residues were depicted as diamonds instead of circles (Figure 3). Especially mutations of residues buried in the structure or close to the functional sites might have a relatively strong impact on protein stability and function. Different node coloring schemes were prepared to map the different types of structural and functional information. This allowed us to identify relevant mutations with functional effects.

In the default secondary structure-colored view, we observed that most mutations are located on the surface of the protein, i.e., in helices (51 out of 100) and loops (45 out of 100), rather than in the interior consisting of strands (only 4) (Figure 3). The conservation-colored view indicated that residues in the protein exterior tend to be more variable in contrast to the ones in the interior where the active site of the enzyme is located (Figure 5). Thus, we could conclude from the visualizations that most mutations are located in more variable regions on the surface of the protein. Thus, mutated residues with strong conservation (F11, L13, Q82, I83, I109, K134, K135, L174, A175, D180, A212, N213, V226) might be responsible for the functional deficit of the mutant (Figure 5).

**Figure 7:**
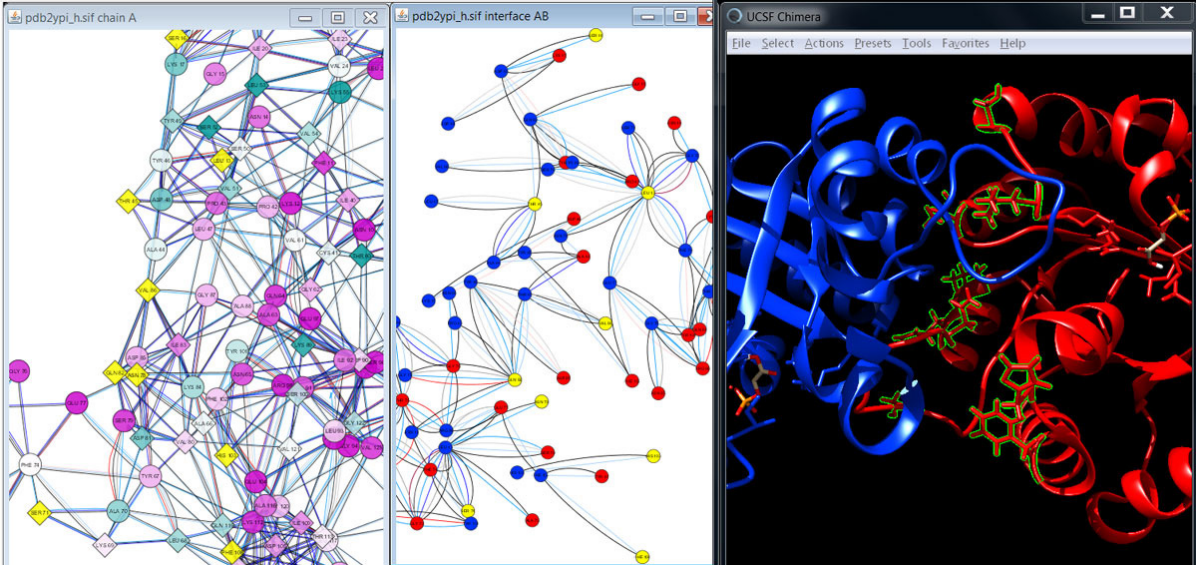
**Visualization of the dimer interface with focus on the mutated residues**. The combined visualization of the conservation-colored RIN of chain A of scTIM (left), the residue nodes in the interface between chain A (red) and chain B (blue) of scTIM (middle), and the ribbon representation of scTIM are in the same colors as provided by UCSF Chimera (right). Mutations located in the dimer interface (V86, T45, S71, S16, Q82, N78, L13, H103, F108) are highlighted by yellow colored nodes in the network views and by green boundaries and ball-and-stick representations in the structure view. Nodes that correspond to mutated residues are depicted as diamonds.

Since scTIM functions as a dimer, another important aspect is the binding interface between the two monomers. We used RINalyzer to extract the residue interactions of the interface and visualized them in a separate network view. As can be seen in Figure 7, 9 out of the 69 residues are mutated (L13, S16, T45, S71, N78, Q82, V86, H103, F108). These changes might impair the dimer formation and thus affect the function of scTIM. Residue L13 is particularly interesting as it is both conserved and in the dimer interface. A similar analysis can be performed with other functional sites. For instance, we found that none of the residues in the active or substrate binding site (N10, K12, H95, E165) are mutated. However, 24 residues possess direct non-covalent interactions with functionally important residues and thus could have a severe impact on their function if mutated. This is the case for the residues F11, L13, and C41, and this observation is further strengthened by the fact that the first two of them are conserved as described above.

**Figure 8:**
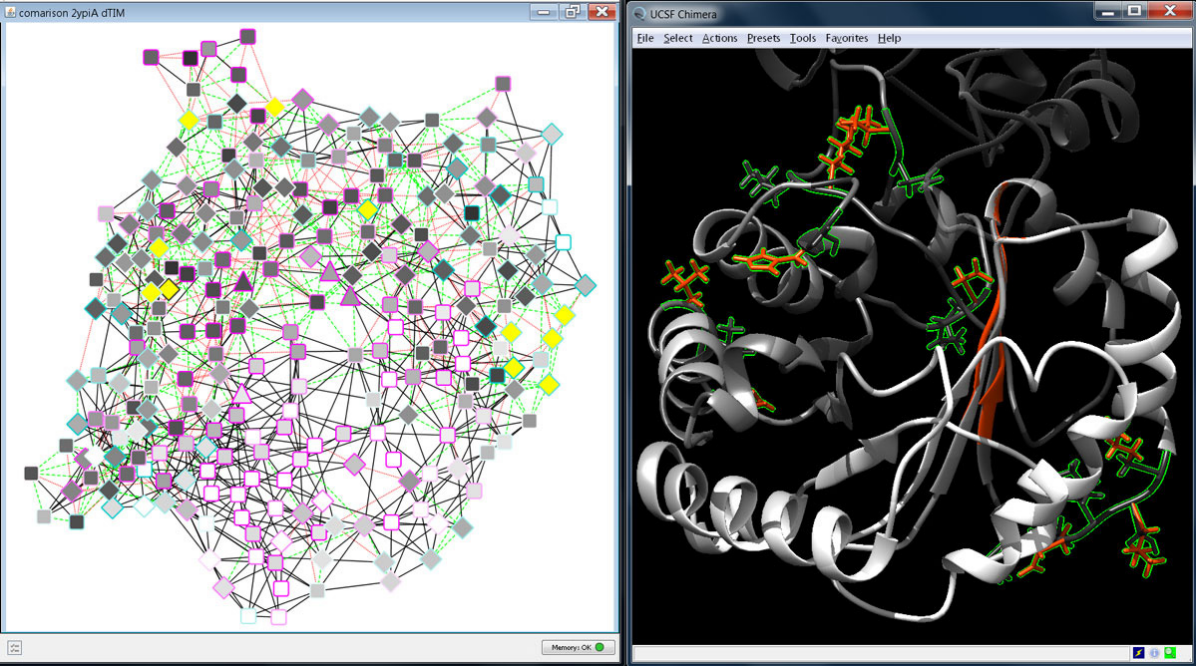
**Highlighted mutations with important impact on residue interactions**. A comparison network is shown in Cytoscape (left) and a visualization of the aligned structures (scTIM in gray, dTIM in red) in UCSF Chimera (right). In the network view, green dashed edges depict gained, and red dotted edges lost interactions. The network nodes are colored according to the fraction of adjacent interaction edges that do not change upon mutation (from white for all to gray for none), the node border colors represent the conservation score of the respective residue in the parent with turquoise-to-pink coloring for variable-to-conserved sites. Nodes with an amino acid mutation are shown as diamonds. The mutated residues with the largest impact on the residue interactions are highlighted by yellow colored nodes in the network views and by green boundaries and the ball-and-stick representations in the structure view. The mutations correspond to the following residue pairs based on the alignment of scTIM (chain A) and dTIM sequences: (A30, -), (S31, K30), (I32, L31), (E34, D33), (N35, D34), (L68, K67), (N78, I77), (K89, D88), (S100, H99), (V154, L153), (-, E156).

The comparison network view provided further information about the location and nature of the mutations (Figure 8). From the overall distribution of red and green edges that indicate changes of non-covalent interactions, it is apparent that many mutations lead to a large number of differences primarily on the protein surface. Additionally, the active site residues form different interactions with their neighbors in the parent compared with the mutant structure. Furthermore, there is an insertion (E156 in dTIM) and a deletion (A30 in scTIM) in the dTIM sequence in contrast to the parent sequence according to the sequence alignment in UCSF Chimera. However, they are not close to the active site or the dimer binding interface and thus the functional effect is difficult to judge. Finally, the residue nodes in Figure 8 are colored according to the fraction of interactions they gained or lost upon mutation.When combining this information with the conservation scores mapped to the node border colors, particularly interesting mutations can be found. Mutations with the largest change of local residue interactions are highlighted in Figure 8 (A30, S31, I32, E34, N35, L68, N78, K89, S100, V154 in scTIM and E156 in dTIM), and the mutated residue with a high conservation score (N78) is especially conspicuous.

By combining the different views and data in an interactive fashion, it became possible to pinpoint a number of residue mutations as candidates for having a pronounced effect on the enzymatic activity of dTIM. Further experimental validation will be needed to determine which mutations have to be replaced in the mutant by amino acids from the parent to rescue functionality. Other structural properties such as hydrophobicity, solvent accessible surface area or polarity can also be mapped onto the RIN view to characterize mutations with particular properties. Another strategy described in our previous work [18] would be the application of network topology analysis of the RIN for the detection of important residues.

## Conclusions

We presented a novel approach for the integrative visual analysis of protein sequence mutations. We extended several existing software tools and combined different visualizations in such a way that biological information can be exchanged between them and additional external data can be included. We also devised a new layout algorithm for the RINs provided by the RINalyzer app in Cytoscape. Additionally, we created a new aggregation network view, improved and enriched the existing comparison network view, incorporated an interface to the Pro-origami web service, and fully utilized the interface to the UCSF Chimera tool through the structureViz app.

In the future, to assess the usefulness and effectiveness of our approach and to improve the current implementation, we intend to collect more user feedback. This will result in a comprehensive evaluation which visual cues are best suited for gaining insight into the impact of mutations, how they should be best mapped onto the sequence, structure, and network representations, and how they should be integrated into the visual layout. Another issue is the aggregation of network regions to reduce the visual complexity as only some of them might be of actual interest to assess the potential impact of mutations. In this way, patterns of mutations with specific functional consequences might become more apparent, in particular, when multiple proteins are analyzed.

We also plan to improve the software integration of the different tools such that our approach can be realized in an automated fashion. This includes better synchronization over linked views and automated retrieval of external data.

## Competing interests

The authors declare that they have no competing interests.

## Authors' contributions

NTD and KK drafted the paper. NTD, KK, MA, and FSD were involved in the design of the project. NTD, KK, JHM, and MW carried out the implementation. All authors edited, read and approved the manuscript.

## Declarations

Publication costs were covered by the Resource for Biocomputing, Visualization, and Informatics at the University of California, San Francisco, the School of Information Technologies at The University of Sydney (Tom Sawyer ARC Grant), and the Max Planck Society.

This article has been published as part of *BMC Proceedings *Volume 8 Supplement 2, 2014: Proceedings of the 3rd Annual Symposium on Biological Data Visualization: Data Analysis and Redesign Contests. The full contents of the supplement are available online at http://www.biomedcentral.com/bmcproc/supplements/8/S2
